# Effectiveness of Urate-Lowering Therapy for Renal Function in Patients With Chronic Kidney Disease: A Meta-Analysis of Randomized Clinical Trials

**DOI:** 10.3389/fphar.2022.798150

**Published:** 2022-03-17

**Authors:** Xiuping Liang, Xiang Liu, Duohui Li, Wei Qin, Yi Liu

**Affiliations:** ^1^ Department of Rheumatology and Immunology, Department of Medicine, West China Hospital, Sichuan University, Chengdu, China; ^2^ West China School of Medicine, Sichuan University, Chengdu, China; ^3^ Department of Nephrology, Department of Medicine, West China Hospital, Sichuan University, Chengdu, China; ^4^ Rare Diseases Center, Institute of Immunology and Inflammation, Frontiers Science Center for Disease-related Molecular Network, West China Hospital, Sichuan University, Chengdu, China

**Keywords:** ULT, chronic kidney disease, hyperuricemia, renal function, meta-analysis

## Abstract

**Background and Objective:** Hyperuricemia is closely related to chronic kidney disease (CKD). The effects of urate-lowering therapy (ULT) on renal outcomes are uncertain, and whether it is warranted in CKD patients is currently unclear. The aim of our meta-analysis of randomized clinical trials (RCTs) was to assess the effectiveness and safety of ULT for improving kidney function in patients with CKD.

**Methods:** RCTs were retrieved from the PubMed, Embase, MEDLINE and Cochrane Central Register of Controlled Trials databases. The meta-analysis was performed using Review Manager and Stata/SE software. The outcomes were changes in renal function and serum uric acid (SUA), serum creatinine, and adverse events.

**Results:** Twelve RCTs with 1,469 participants were included in the meta-analysis. ULT was found to effectively lower SUA (standard mean difference (SMD): -2.70; 95% confidence interval (CI): -3.71, -1.69) but the renoprotective effects were not superior to those of control therapy (placebo or usual therapy), which were stable in the subgroup analyses and sensitivity analyses. Regarding adverse events, their risks did not increase in the ULT group compared with the control group and were stable in the sensitivity analyses.

**Conclusion:** The findings of our meta-analysis suggested that ULT can effectively lower SUA, but there is insufficient evidence to support the renoprotective effects of ULT in CKD patients. In addition, ULT is safe for patients with CKD.

**Systematic Review Registration:** https://clinicaltrials.gov/, identifier PROSPERO (CRD42020200550).

## Introduction

Chronic kidney disease (CKD) is defined as abnormalities in kidney structure or function that are present for more than 3 months and have an impact on health. These abnormalities include a decreased glomerular filtration rate and increased urinary albumin-creatinine ratio (Stevens and Levin, 2013). CKD progression can lead to end-stage renal disease and other organ damage, such as cardiovascular events, which highlights the need to adopt more effective treatment strategies for CKD (2). Hyperuricemia is a condition commonly defined as a serum uric acid (USA) concentration over 5.7 mg/dl for women and 6.8 mg/dl for men. This may be caused by excessive production of uric acid or its reduced excretion (Eleftheriadis et al., 2017; Dalbeth et al., 2019). The kidneys have a strong relationship with uric acid, as most daily uric acid excretion (65–75%) occurs by reabsorption and secretion in the proximal tubule (Cannella and Mikuls, 2005; Preitner et al., 2009; Wright et al., 2010; Bobulescu and Moe, 2012). Therefore, hyperuricemia is associated with impaired renal excretion of uric acid (Richette and Bardin, 2010).

Hyperuricemia is also closely related to CKD, but the causality is still not very clear (2). Previous studies suggested that SUA was likely a risk factor for CKD (Xiao et al., 2015; Crișan et al., 2016; Eleftheriadis et al., 2017). At present, there are many urate-lowering drugs, including xanthine oxidase inhibitors (allopurinol and febuxostat), pegloticase, interleukin-1 (IL-1) blockers, and lesinurad (URAT1 inhibitor). However, whether the use of these drugs can postpone CKD progression and whether it is necessary for CKD patients to undergo ULT is still unclear (2). Further evidence is needed to define the efficacy and safety of ULT in CKD patients.

Thus, we performed a systematic review and meta-analysis of RCTs to evaluate the efficacy and safety of ULT in patients with CKD.

## Methods

### Data Sources and Literature Search

This systematic review was performed according to the Cochrane Handbook for Systematic Reviews of Interventions and the Preferred Reporting Items for Systematic Reviews and Meta-Analysis (PRISMA) statement (Moher et al., 2009). It was registered with PROSPERO (CRD42020200550). RCTs were retrieved from the PubMed, Embase, MEDLINE and Cochrane Central Register of Controlled Trials databases up to 3 August 2020. The search strategy included the following key words: “renal insufficiency, chronic”, “CKD”, “hyperuricemia”, “ULT”, “febuxostat”, “allopurinol”, “probenecid”, “rasburicase”, “pegloticase”, and “benzbromarone”. In addition, systematic reviews, guidelines, and reference lists from all relevant articles were also manually searched to identify potential eligible studies. Ethical approval was not necessary because our study was a meta-analysis.

### Inclusion and Exclusion Criteria

Studies were included in our meta-analysis if they met the following criteria: 1) RCTs involved patients with CKD; 2) the dosage and duration of the intervention (ULT) and control groups (placebo or usual therapy) were adequately documented; and 3) changes in uric acid, estimated glomerular filtrate rate (eGFR), creatinine, or proteinuria were reported. Studies were excluded if they 1) lacked a control group (placebo or usual therapy), 2) were not published in English, 3) had a follow-up time <3 months. Study selection was performed independently by two authors (XPL and DL). Any disagreement was resolved by the third reviewer (XL).

### Outcomes

Changes in the USA, eGFR, serum creatinine and adverse events including deterioration of kidney function, liver dysfunction, cardiovascular events, gastrointestinal symptoms, and hypersensitivity (such as rash) were assessed in our article.

### Data Extraction and Quality Assessment

Two researchers (XPL and DL) independently extracted the information, including title, first author, year of publication, study design, country of origin, inclusion and exclusion criteria, sample size, study population characteristics, drug type and dosage, intervention period and duration, outcomes, and adverse events. Outcomes including adverse events, changes in SUA, serum creatinine, and eGFR were used to evaluate the effects and safety of ULT. When the above data were unavailable, they were extracted from figures and/or tables as far as possible. Any inconsistency between the two researchers was resolved by the third researcher (XL) to reach an agreement.

The risk of bias of the included RCTs was assessed independently by two researchers (XPL and XL) according to the revised Cochrane risk-of-bias tool for randomized trials (Higgins et al., 2011). The risk-of-bias assessment consisted of random sequence generation, allocation concealment, participant blinding, investigator binding, incomplete outcome data, selective reporting, and other bias. Judgment of the risk of bias arising from each domain was generated by an algorithm. The risk of bias was categorized as high, low, or unclear.

### Data Synthesis and Statistical Analysis

We completed all statistical analyses by the statistical package Review Manager and Stata/SE software. All pooled data of eGFR, SUA, creatinine and blood pressure, as continuous variables, are shown with SMD and 95% CI. The pooled data of adverse events, as binary variables, were evaluated with risk ratios (RRs) and 95% CIs. We considered that *p* < 0.05 indicated a significant difference. We used the Q-test and I statistic to examine heterogeneity. If studies were heterogeneous (*p* < 0.1 or I^2^ > 50%), the random-effects model was used. Otherwise, the fixed-effects model was applied (Liu et al., 2021). We also used leave-one-out sensitivity analysis (Liu et al., 2018) and subgroup analyses to explore heterogeneity, including CKD stages, follow-up time and different uric acid-lowering agents. In addition, Egger’s test of publication bias was performed with Stata/SE software, and *p* < 0.05 indicated the presence of publication bias.

## Result

### Search Results and Characteristics of the Studies

**FIGURE 1:**
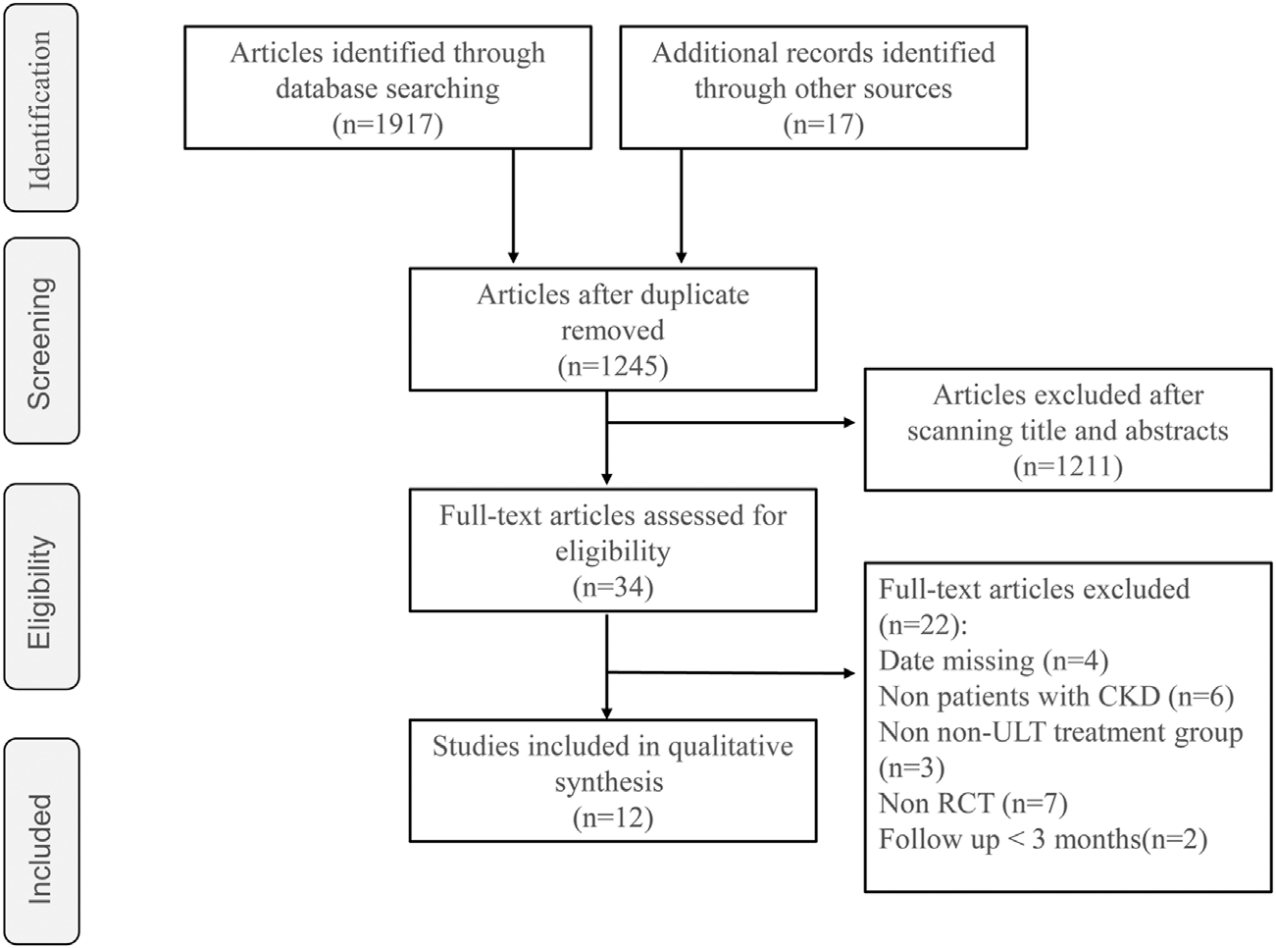
Flowchart of the study selection procedure.

A total of 1,245 studies were identified during the initial search, and we eliminated 1,211 studies based on our screening of titles and abstracts. After reading the full texts of 34 studies in detail, 12 RCTs with 1,469 participants were included in this meta-analysis (Figure 1)

The essential information of the 12 RCTs is shown in Table 1 and Table 2. All participants in the 12 RCTs (Siu et al., 2006; Goicoechea et al., 2010; Momeni et al., 2010; Kao et al., 2011; Shi et al., 2012; Sircar et al., 2015; Saag et al., 2016; GhaneSharbaf and Assadi, 2018; Kimura et al., 2018; Mukri et al., 2018; Badve et al., 2020; Stack et al., 2021)were CKD patients, 6 RCTs (15, 16, 19-21, 24) incorporated patients with hyperuricemia, while the level of SUA was not clearly reported in the other 6 studies (17, 18, 22, 23, 25, 26). The ULT drugs included febuxostat in seven studies (15-19, 22, 25) and allopurinol in five studies (20, 21, 23, 24, 26), while the control group was placebo or usual therapy. The follow-up period ranged from 4 to 27 months.

**TABLE 1:** study characteristics of included studies.

Author/Year	Country	Study type	Samples (ULT/CTL)	Intervention (mg/d) controls	Follow up (months)	Inclusion criteria
Siu et.al., 2006	China	RCT	25	Allopurinol (100-200)	12	SUA≥7.60 mg/dl proteinuria>500 mg/d and/or SCr>120 μmol/L
			26	Usual therapy		
Goicoechea et.al., 2010	Spain	RCT	57	Allopurinol (100)	23.4 ± 7.8	Mean SUA: 7.6 mg/dl eGFR <60 ml/min /1.73 m^2^
			56	Usual therapy		
Ali et.al 2010	British	RCT	20	Allopurinol (100) placebo	4	Mean SUA: 6.23 mg/dl
			20			Diabetic nephropathy
Kao et.al., 2011	British	RCT	27	Allopurinol (300)	9	Mean SUA: 7.23 mg/dl
			26	Usual therapy		CKD 3
Shi et.al., 2012	China	RCT	21	Allopurinol (100-300) placebo	6	SUA> 6 mg/dl (women), SUA> 7 mg/dl (men)
			19			IgA nephrology
Sircar et.al., 2015	India	RCT	45	Febuxostat (40) placebo	6	SUA >7 mg/dl
			48			CKD 3,4
Saag et.al., 2016	United States	RCT	32	Febuxostat (40-80) placebo	12	SUA >7.0 mg/dl eGFR 15–50 ml/min /1.73 m^2^
			32			
Kimura et.al., 2018	Japan	RCT	219	Febuxostat (10-40) placebo	27	SUA: 7.0–10 mg/dl, CKD 3
			222			
Ghane et.al 2018	Iran	RCT	38	Allopurinol (5 mg/kg and <300 mg)	4	SUA> 5.5 mg/dl
			32	Usual therapy		CKD 1–3
Mukri et.al., 2018	Malaysia	RCT	47	Febuxostat (40)	6	SUA> 7.60 mg/dl
			46	Usual therapy		CKD 3,4
Badve et.al., 2020	Australia	RCT	176	Allopurinol (100-300) placebo	26	CKD 3,4
			175			
Stack et.al., 2021	British	RCT	32	Febuxostat (80) and verinurad (Crișan et al., 2016)	6	SUA> 6.0 mg/dl
			28	placebo		Diabetic nephropathy

Abbreviations: RCT, randomized controlled trial; SUA, serum uric acid; CKD, chronic kidney disease; eGFR (ml/min/1.73 m^2^), estimated glomerular filtration rate; SCr, serum creatinine; ULT, urate-lowering therapy; CTL, control.

**TABLE 2:** Baseline characteristics of included patients.

Author/Year	Participants	Participants (ULT/CTL)	Gender (% Male)	Mean age	Baseline SUA:(mg/dl)	Baseline kidney function
Siu et.al., 2006	51	25	16%	47.7 ± 12.9	9.75 ± 1.18	Proteinuria 2.39 ± 2.88 g/d
		26	57.7%	48.8 ± 16.8	9.92 ± 1.68	2.39 ± 2.2 g/d
Goicoechea et.al., 2010	113	57	—	72.1 ± 7.9	7.8 ± 2.1	eGFR 40.8 ± 11.2
		56		71.4 ± 9.5	7.3 ± 1.6	39.5 ± 12.4
Ali et.al 2010	40	20	45%	56.3 ± 10.6	5.96 ± 1.21	SCr 1.3 ± 0.45
		20	45%	59.1 ± 10.6	6.5 ± 2.2	1.5 ± 0.6
Kao et.al., 2011	53	27	59.3%	70.6 ± 6.9	7.39 ± 1.51	eGFR 44 ± 11
		26	46.2%	73.7 ± 5.3	7.06 ± 1.34	46 ± 9
Shi et.al., 2012	40	21	61.9%	39.7 ± 10	7.9 ± 1.1	eGFR 69.5 ± 26.5
		19	47.4%	40.1 ± 10.8	7.8 ± 1.1	63.6 ± 27.5
Sircar et.al., 2015	93	45	64.4%	56.22 ± 10.87	9 ± 2	eGFR 31.5 ± 13.6
		48	77.1%	58.42 ± 14.52	8.2 ± 1.1	32.6 ± 11.6
Saag et.al., 2016	64	32	79.7%	65.51 ± 9.84	10.36 ± 1.56	eGFR 34.1
		32	81.3%	66.3 ± 12.05	10.8 ± 1.96	29.31
Kimura et.al., 2018	441	219	77.6%	65.3 ± 11.8	7.8 ± 0.9	eGFR 45.2 ± 9.5
		222	77.0%	65.4 ± 12.3	7.8 ± 0.9	44.9 ± 9.7
Ghane et.al 2018	70	38	55.3%	6.3 ± 2.9	6.7 ± 1.2	eGFR 76.2 ± 12.7
		32	53.1%	6.2 ± 2.7	6.5 ± 1.3	79.2 ± 13.1
Mukri et.al., 2018	93	47	53.2%	64 (10)	9.07 ± 1.75	eGFR 26.2 ± 14.3
		46	54.3%	67 (6)	9.03 ± 1.19	28.2 ± 19.8
Badve et.al., 2020	351	182	61.5%	62.3 ± 12.6	8.2 ± 1.8	eGFR 31.6 ± 11.7
		181	64.1%	62.6 ± 12.9	8.2 ± 1.7	31.9 ± 12.4
Stack et.al., 2021	60	32	69%	—	7.5 ± 1.6	eGFR 59.2 ± 25.3
		28	71%		7.0 ± 0.8	68.1 ± 23.2

Abbreviations: eGFR (ml/min/1.73 m^2^), estimated glomerular filtration rate; SCr (mg/dl), serum creatinine; SUA, serum uric acid; ULT, urate lowering therapy; CTL, control.

### Effect of ULT on SUA

**FIGURE 2:**
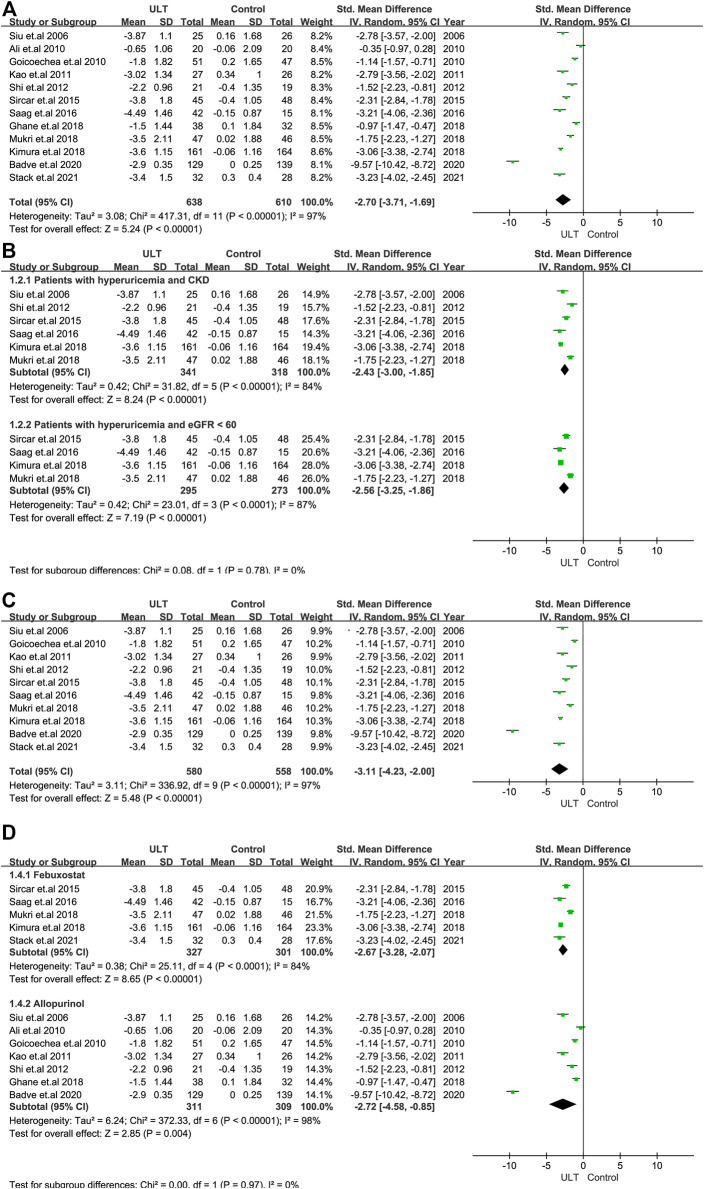
The effect of ULT compared with the control on SUA. **(A)** All patients. **(B)** Subgroup analysis of patients with hyperuricemia and CKD or patients with hyperuricemia and eGFR <60 ml/min/1.73 m^2^. **(C)** Subgroup analysis including patients with a treatment duration of 6 months or over. **(D)** Subgroup analysis for ULT drugs including febuxostat and allopurinol. Abbreviations: SMD, standard mean difference; CI, confidence interval; eGFR, estimated glomerular filtration rate.

Twelve RCTs with 1,248 patients showed that ULT significantly decreased the level of SUA compared to the control group (SMD: -2.70; 95% CI: -3.71, -1.69) (Figure 2A). In the analysis of data with only patients with CKD and hyperuricemia or patients with eGFR<60 ml/min/1.73 m^2^ and hyperuricemia included, the results were consistent. (Figure 2B).

We performed subgroup analysis according to the treatment duration (6 months) and the ULT drugs, including febuxostat and allopurinol. The analysis of studies with treatment durations of 6 months or more showed the consistent results above (Figure 2C), as did the analysis of studies including only febuxostat or allopurinol (Figure 2D).

### Effect of ULT on Kidney Outcomes

**FIGURE 3:**
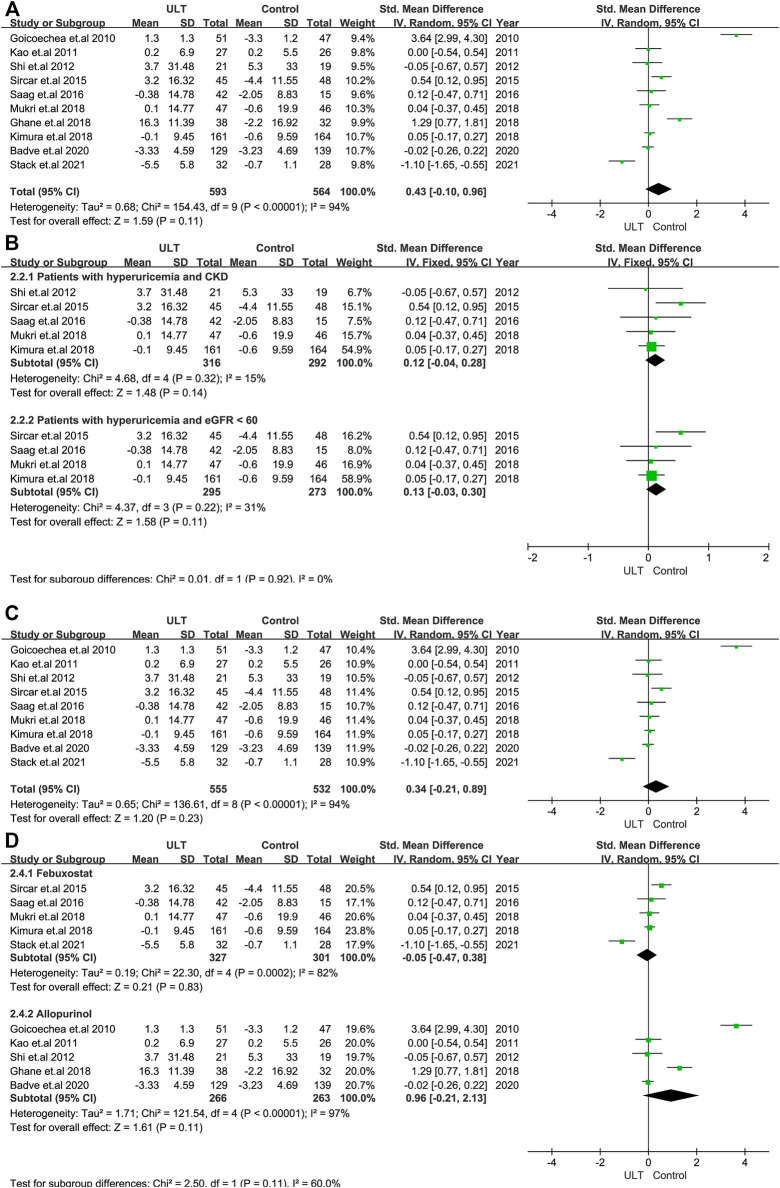
The effect of ULT compared with the group on eGFR **(A)** All patients. **(B)** Subgroup analysis of patients with hyperuricemia and CKD or patients with hyperuricemia and eGFR <60 ml/min/1.73 m^2^. **(C)** Subgroup analysis including patients with a treatment duration of 6 months or over. **(D)** Subgroup analysis for ULT drugs including febuxostat and allopurinol. Abbreviations: SMD, standard mean difference; CI, confidence interval; eGFR, estimated glomerular filtration rate.

**FIGURE 4:**
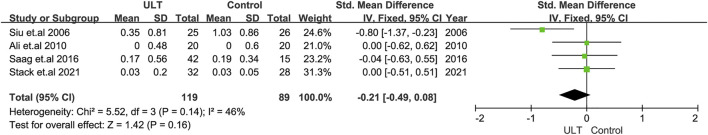
The effect of ULT compared with the control on serum creatine. Abbreviations: SMD, standard mean difference; CI, confidence interval.

Renal function was evaluated by the changes in eGFR and serum creatinine. In terms of the change in the level of eGFR, 10 RCTs with 1,157 patients showed no statistically significant differences between the ULT and control groups (SMD: 0.43; 95% CI: 0.10, 0.96) (Figure 3A). When only patients with CKD and hyperuricemia or patients with eGFR<60 ml/min/1.73 m^2^ and hyperuricemia were included, the changes were still not statistically significant (Figure 3B). The subgroup analysis including nine studies with treatment durations of 6 months or more and the subgroup according to ULT drugs (febuxostat or allopurinol) also demonstrated that there were no differences (Figures 3C,D). Regarding serum creatinine, four studies with 208 patients revealed consistent results with eGFR (Figure 4).

### Safety of ULT

The major concern about febuxostat is its cardiovascular side effect supported by the RCT performed by White et al., which demonstrated that febuxostat had higher all-cause mortality and cardiovascular mortality than allopurinol (White et al., 2018), and this was noticed by the U.S. Food and Drug Administration (FDA). Therefore, we directly performed a subgroup analysis according to febuxostat and allopurinol. Three studies with 501 patients showed that the risk of cardiovascular events was slightly lower in the allopurinol group than in the control group (Risk Ratio (RR) = 0.68: 95% CI: 0.48, 0.97), while four studies with 690 patients demonstrated that febuxostat had no effect on the risk of cardiovascular events (RR = 0.74: 95% CI: 0.36, 1.52) (Supplementary Figure S1). Furthermore, there were no differences in the other adverse events between the ULT and control groups (Supplementary Figure S2).

### Assessment of Risk of Bias and Publication Bias

**FIGURE 5:**
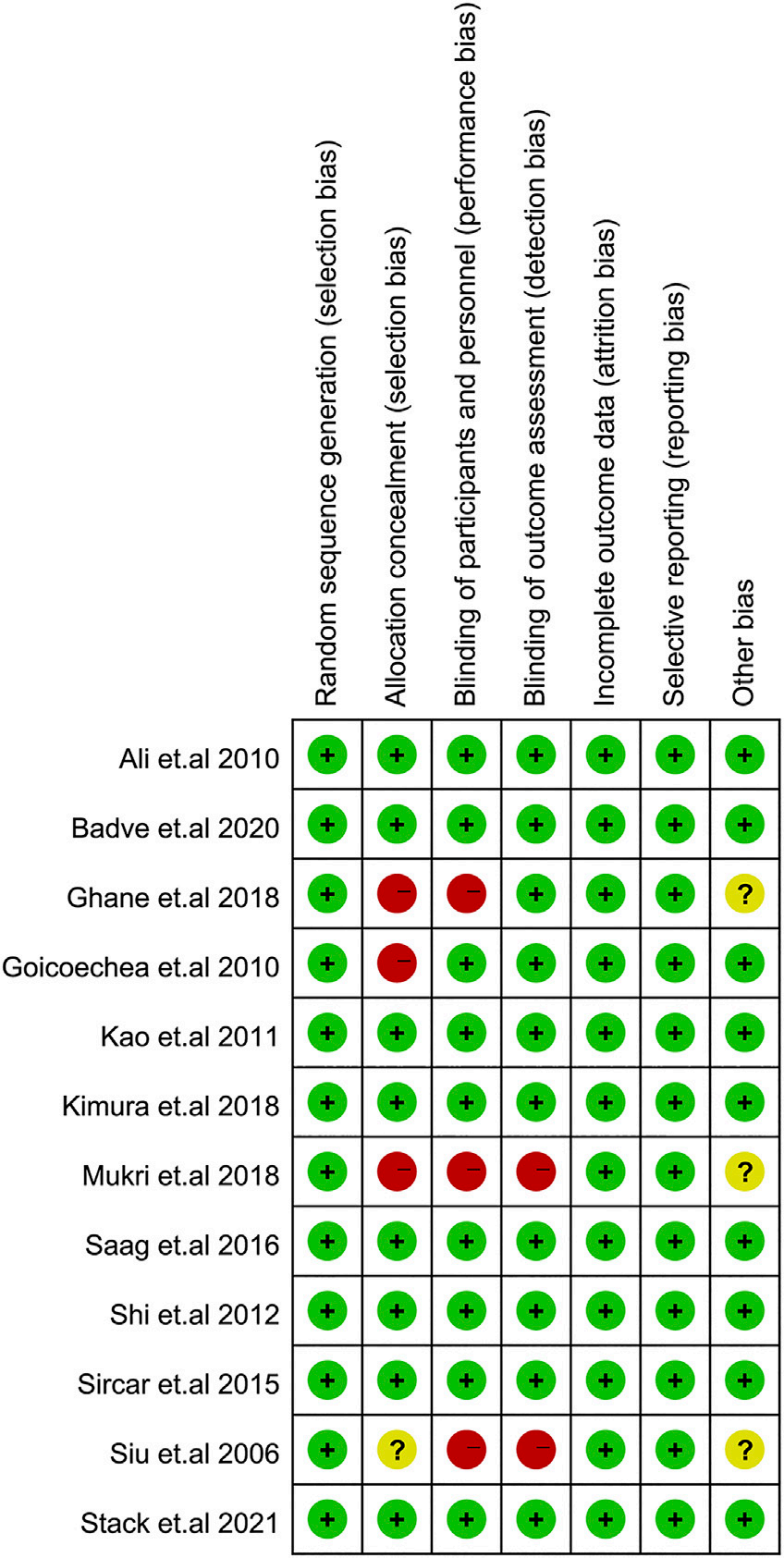
Assessment of risk of bias items for the included studies.

The risk of bias of each study is shown in Figure 5. The overall risk of bias comprised of 8 studies with a low risk (17-21, 23, 25, 26) and 4 studies with a high risk of bias (15, 16, 22, 24), which was mainly due to the risk of participant or investigator blinding.

Egger’s test assessing bias for our main primary outcome (the change in eGFR) was not significant (*p* = 0.312). However, the funnel plot for the outcome of eGFR in this meta-analysis showed asymmetry (Supplementary Figure S3). Therefore, the results may be affected by publication bias.

## Discussion

We performed this meta-analysis to evaluate the efficacy, renoprotective effect, and safety of ULT among patients with CKD. Twelve RCTs with 1,469 participants were included (15-26). ULT has been shown to effectively lower SUA, and the result was consistent in subgroup analyses and the sensitivity analysis. However, ULT was not found to have renoprotective effects, in terms of eGFR or serum creatinine, each of which was stable in the subgroup analyses and sensitivity analyses.

Our study is the largest meta-analysis to evaluate the effectiveness of renal protection between the ULT and control groups. We included 12 RCTs with 1,469 participants in all and ultimately assessed the change in the level of eGFR in 10 RCTs with 1,157 participants, which demonstrated no difference between the ULT and control groups. This was in agreement with some RCTs suggesting that there were no renoprotective effects with ULT. For example, Badve et al. (Badve et al., 2020) performed an RCT across 31 centres with 351 participants published in the New England Journal of Medicine and demonstrated that allopurinol did not ameliorate the renal function in terms of eGFR in patients with CKD. In contrast, ULT was demonstrated to have renoprotective effects in meta-analyses performed by Wang et al. (Wang et al., 2013), Kanji et al. (Kanji et al., 2015) and Su et al. (Su et al., 2017). However, the studies by Wang et al. and Kanji et al. both analyzed trials with short follow-up times (less than 2 months). The three studies all incorporated several non-English RCTs and assessed eGFR with fewer RCTs than our study. Owing to these contradictory results, large-scale RCTs of high quality are needed to explore the effects of ULT on renal function, especially in patients with CKD.

At present, many studies including clinical and mechanistic research, have explored the relationships among uric acid, cardiovascular diseases and the effect of ULT, and the causality among these variables is very controversial. A cross-sectional study in patients with unilateral small kidney or renal agenesis conducted by Yazici et al. demonstrated that the SUA level was positively correlated with arterial stiffness (Yazici et al., 19932017). Mechanistic studies have shown that uric acid causes endothelial dysfunction, resulting in cardiovascular events (Khosla et al., 2005; Liang et al., 2015; Lee et al., 2021; Singh et al., 2021). In our meta-analysis, we found that allopurinol might slightly reduce the risk of cardiovascular complications in only three RCTs. Therefore, the sample is not enough to draw a conclusion. Large-scale studies are needed. Moreover, there were no differences in the other adverse events between the ULT and control groups. The results above suggest that ULT is at least safe for patients with CKD.

There are some strengths in our review. First, we included only RCTs with a treatment duration of more than 3 months. Second, the number of included RCTs and patients was relatively appreciable. Furthermore, we performed subgroup analysis by CKD stage and treatment duration, and changes in renal function, including eGFR and serum creatinine, were comprehensively evaluated. The adverse effects incorporating deterioration of renal or liver function, cardiovascular events, gastrointestinal symptoms and hypersensitivity were also analyzed. However, our study also has some limitations. First, the risk of bias of 4 studies was high and this was reflected in the risk of participant or investigator blinding. Second, the evaluation of progression to end-stage renal disease was insufficient (no data). Third, the sample sizes of the studies included were quite small, ranging from 40 to 351.

## Conclusion

The findings of our meta-analysis suggest that the evidence is insufficient to support the renoprotective effects of ULT in CKD patients. Furthermore, ULT shows a superior decrease in SUA and ULT is safe for patients with CKD. However, a well-designed, large-scale, controlled study is required for further clarification.

## Data Availability

The original contributions presented in the study are included in the article/Supplementary Material, further inquiries can be directed to the corresponding authors.
