# Supplementary material for: Effectiveness of Urate-Lowering Therapy for Renal Function in Patients With Chronic Kidney Disease: A Meta-Analysis of Randomized Clinical Trials
Source: Front Pharmacol. 2022 Mar 17;13:798150. doi: 10.3389/fphar.2022.798150 (PMC8968869; doi:10.3389/fphar.2022.798150)
Supplement: Supplementary file 1 [file Table1.DOCX]

Supplementary Figure 1: The effect of ULT compared with the control on the risk of cardiovascular events by subgroup analysis according to ULT drugs including febuxostat and allopurinol.

Supplementary Figure 2: The safety of ULT compared with the control on deterioration of renal function (A), deterioration of liver function(B), gastrointestinal symptoms (C)and hypersensitivity(D).

Supplementary Figure 3: The funnel plot for the outcome of eGFR.
