# Supplementary figures and images for: Effectiveness of Urate-Lowering Therapy for Renal Function in Patients With Chronic Kidney Disease: A Meta-Analysis of Randomized Clinical Trials
Source: Front Pharmacol. 2022 Mar 17;13:798150. doi: 10.3389/fphar.2022.798150 (PMC8968869; doi:10.3389/fphar.2022.798150)

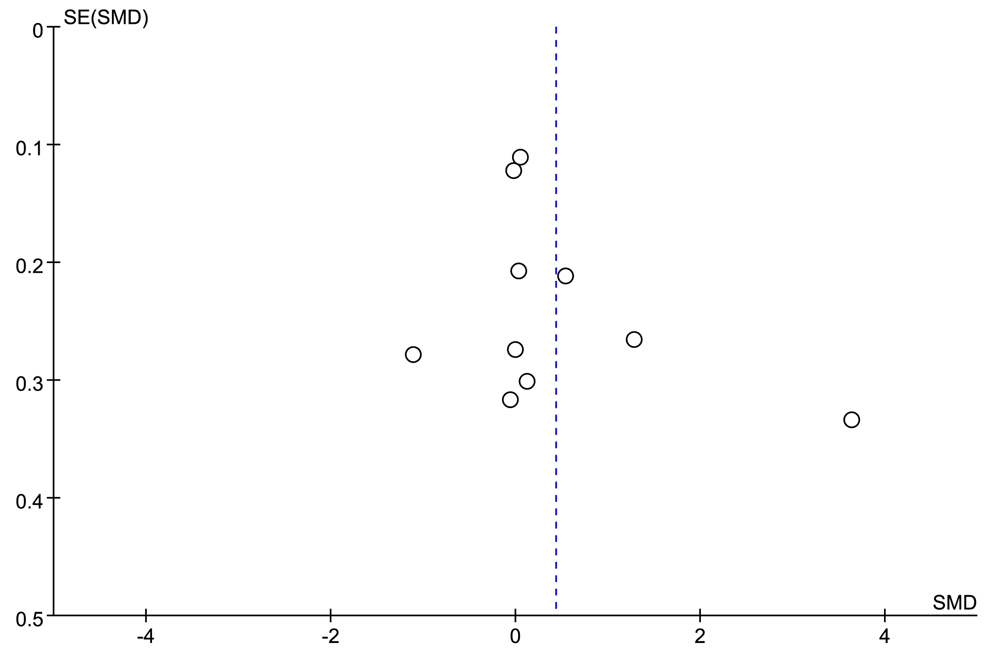

Supplement: Supplementary file 2 [file Image3.TIF]

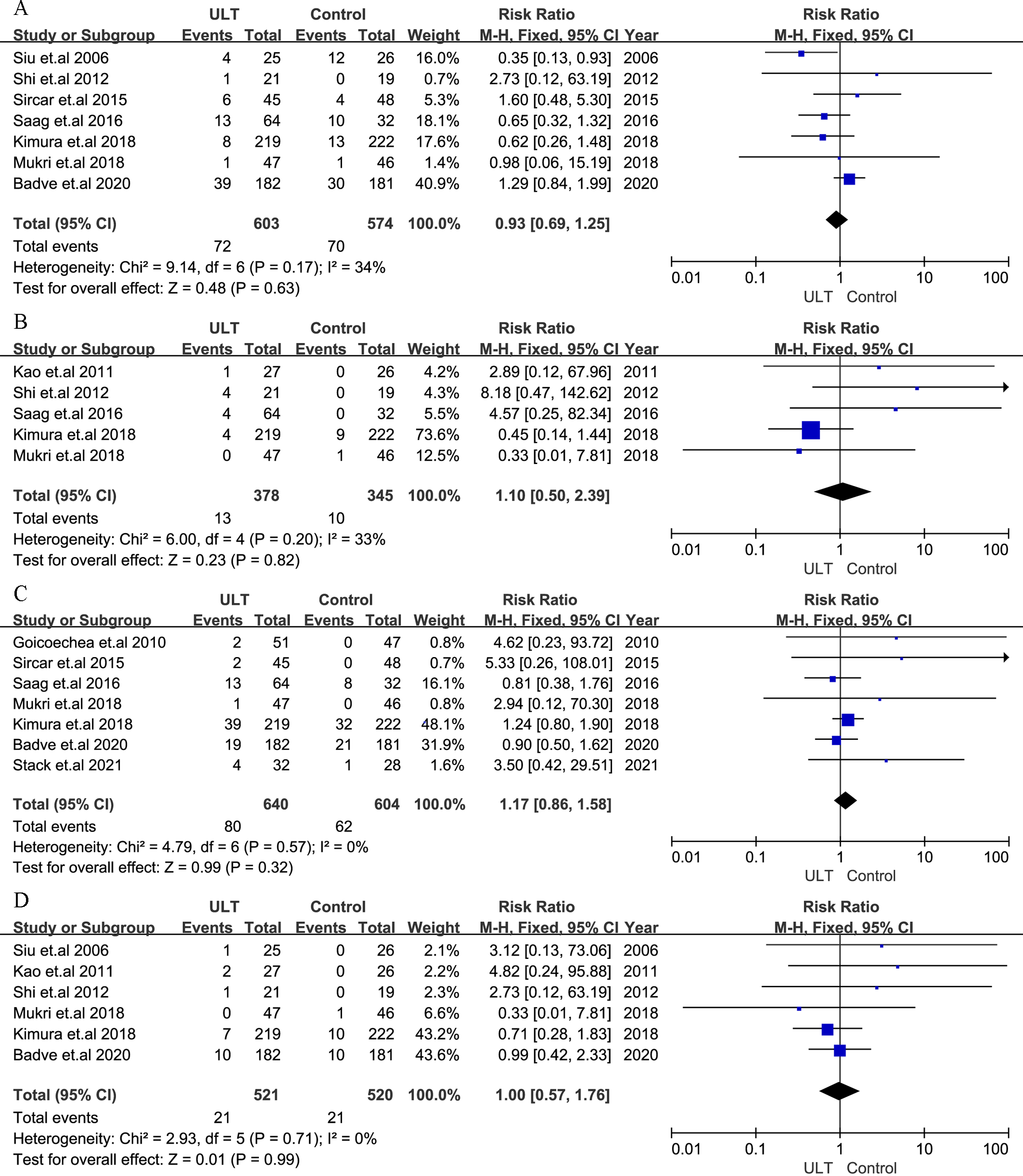

Supplement: Supplementary file 3 [file Image2.TIF]

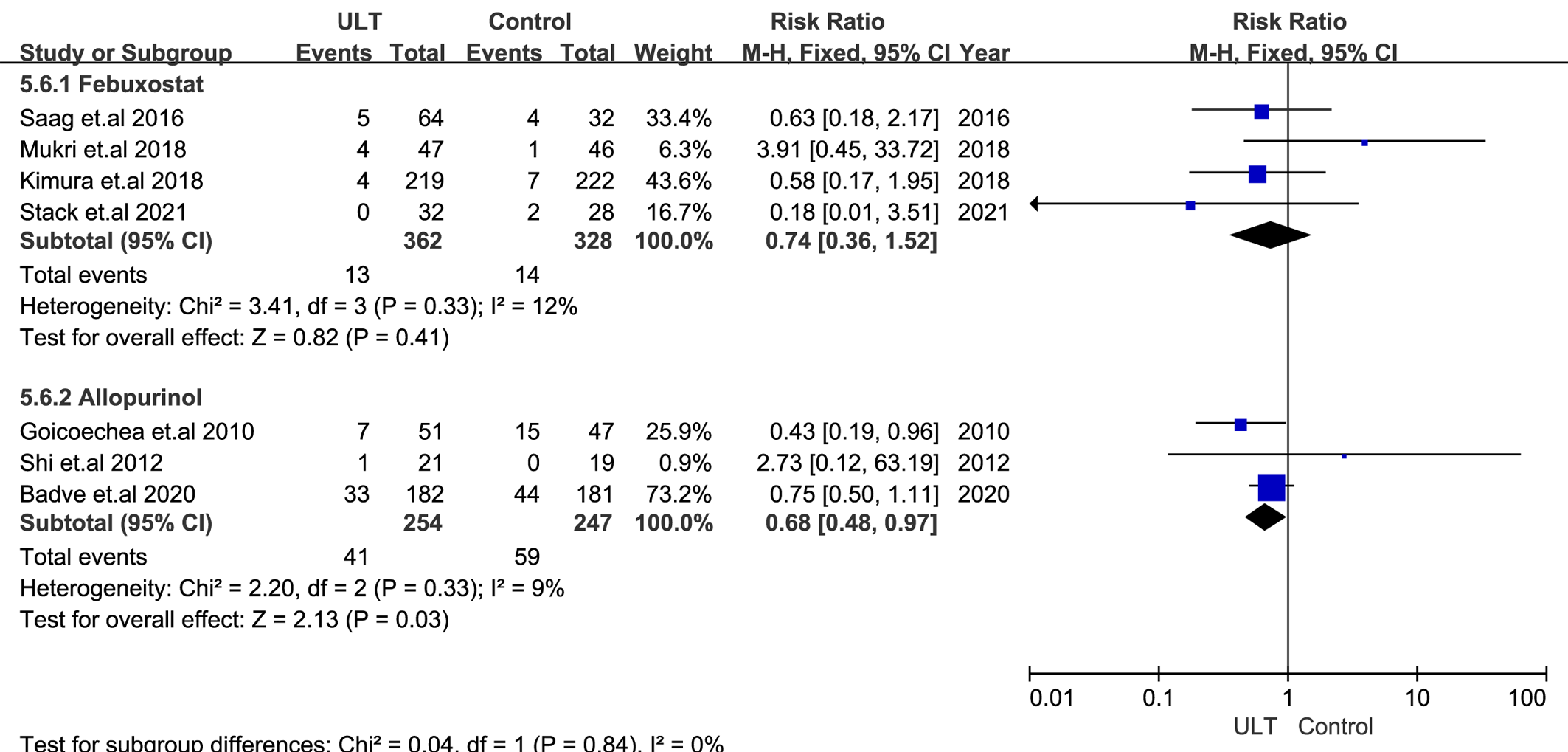

Supplement: Supplementary file 4 [file Image1.TIF]
